# AlignStatPlot: An R package and online tool for robust sequence alignment statistics and innovative visualization of big data

**DOI:** 10.1371/journal.pone.0291204

**Published:** 2023-09-20

**Authors:** Alsamman M. Alsamman, Achraf El Allali, Morad M. Mokhtar, Khaled Al-Sham’aa, Ahmed E. Nassar, Khaled H. Mousa, Zakaria Kehel

**Affiliations:** 1 Agricultural Genetic Engineering Research Institute, Giza, Egypt; 2 African Genome Center, Mohammed VI Polytechnic University, Ben Guerir, Morocco; 3 International Center for Agriculture Research in the Dry Areas, Giza, Egypt; University College London Institute of Ophthalmology, UNITED KINGDOM

## Abstract

Multiple sequence alignment (MSA) is essential for understanding genetic variations controlling phenotypic traits in all living organisms. The post-analysis of MSA results is a difficult step for researchers who do not have programming skills. Especially those working with large scale data and looking for potential variations or variable sample groups. Generating bi-allelic data and the comparison of wild and alternative gene forms are important steps in population genetics. Customising MSA visualisation for a single page view is difficult, making viewing potential indels and variations challenging. There are currently no bioinformatics tools that permit post-MSA analysis, in which data on gene and single nucleotide scales could be combined with gene annotations and used for cluster analysis. We introduce “AlignStatPlot,” a new R package and online tool that is well-documented and easy-to use for MSA and post-MSA analysis. This tool performs both traditional and cutting-edge analyses on sequencing data and generates new visualisation methods for MSA results. When compared to currently available tools, AlignStatPlot provides a robust ability to handle and visualise diversity data, while the online version will save time and encourage researchers to focus on explaining their findings. It is a simple tool that can be used in conjunction with population genetics software.

## Background

Multiple sequence alignment (MSA) is fundamental to the study of genetic variations leading to phenotypic variations in all living organisms. It can be used to identify sequence regions that lead to differences in gene structure and thus gene functionality. MSA analysis is used to study inter- and intra-diversity in order to understand the population structure of the collected DNA samples, which may indicate the origins of evolution and emergence of species [1]. Several dynamic programming algorithms [2] have been used in a variety of programming tools to improve efficiency in various molecular genetic studies. Despite the simple structure of the MSA output, it contains a wealth of information about sequence structure and uniqueness and can be used to extract incomparable information for a wide range of genetic applications. When gene structure and annotation data are combined with MSA results, a more detailed picture of the location of sequence variations in genes emerges, which can be used to assess mutational effects and identify gene functionality. The use of gene annotation with sequence alignment to study susceptibility genes and identify pathogenic mutations is useful in cancer genetics [3]. In crop science, MSA is also applied to identify the molecular basis of biotic or abiotic resistance in cultivated crops, which enables varietal improvement through marker-assisted selection [4]. MSA analysis is useful for evaluating gene classes and gene structures when studying gene families, and the addition of gene annotations helps identify structural domains and functional regions [5]. The MSA results could be converted to advanced genomic data formats such as variant calling format (VCF) or haplotype map (HAPMAP), so that diversity studies and genome-wide association studies can be performed. Few bioinformatics tools such as SNP-sites [6] are used to convert MSA format to VCF format.

Despite the abundance of tools for processing MSA results, there are some challenges that researchers face on a daily basis. MSA visualization tools are extremely useful when dealing with small sets of sequences with short lengths, such as short exons or partial genes. For sequences with tens of thousands of characters or huge datasets, it is difficult to visualize the data on a single page, making it difficult to search for potential indels and variations. Most articles show only a few sequences or parts of the sequences studied to keep plot sizes small. This task has become so overwhelming that tools and pipelines are required to obtain conclusive results and understandable, publication-ready figures. Despite the abundance of tools for processing MSA results, there are some challenges that researchers face on a daily basis. To date, there are no bioinformatics tools that allow post-MSA analysis, where information on sequence variations on genes and SNP scales could be used for cluster analysis and combined with gene annotations. The generation of bi-allelic data and the comparison of wild and alternative gene forms are crucial steps in population genetics. In this paper, we introduce a new R package and web-based tool called “AlignStatPlot”, which is a well-documented MSA and post-MSA analysis tool. This tool generates new visualization techniques for MSA results and performs both traditional and novel analysis techniques on sequencing data. AlignStatPlot is a simple analysis tool that can be combined with population genetics software to help genetic researchers search for genetic variation that controls the manifestation of disease or stress tolerance. In addition, the tool is also freely available online for those who do not want to install the package through R programming language.

## Methods

### Analytical procedure

**Fig 1:**
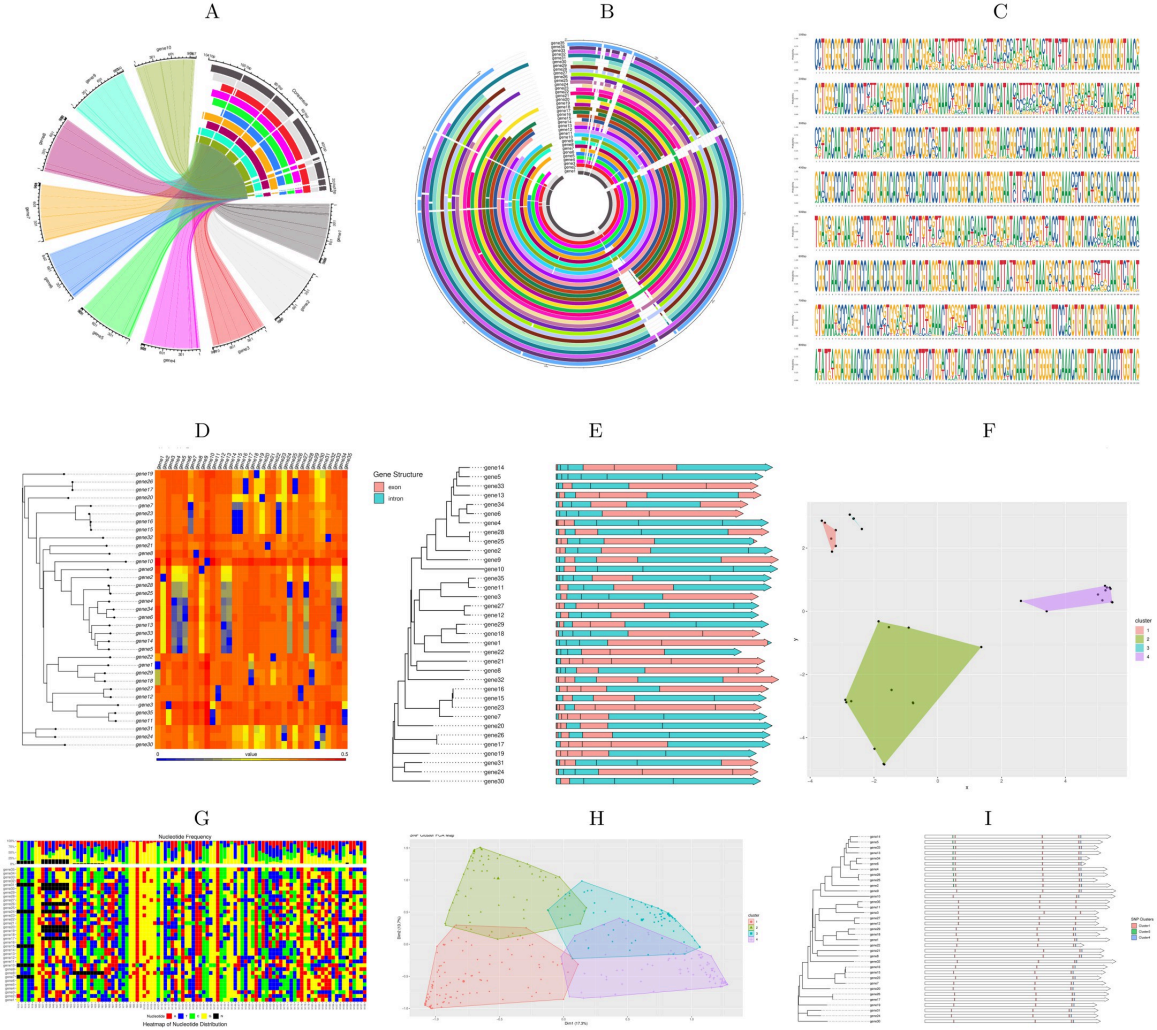
Some sequence alignment statistics visualization were generated using AlignStatPlot, both with online and local tools. The figures include (A) MSA analysis results for a low number of sequences (15 sequences) and (B) for a large number of sequences (15–300 sequences), showing shared regions between aligned sequences. Additionally, (C) displays nucleotide frequency across the MSA, (D) represents the heatmap of the sequence dissimilarity matrix, (E) integrates the phylogenetic tree with sequence annotation, (F) showcases the PCA analysis performed on the studied samples using their sequence variation, and (G) presents nucleotide frequency across the MSA. Furthermore, there is a clustering analysis of MSA-generated SNPs visualized as PCA (H), and their location on gene sequences combined with the phylogenetic tree (I).

**Fig 2:**
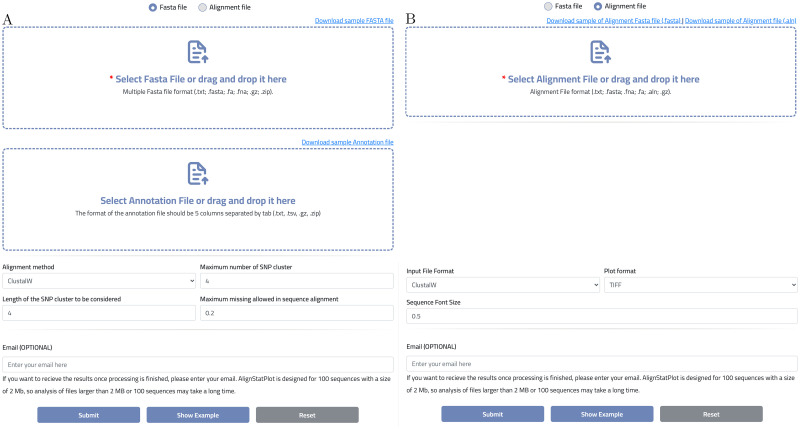
The AlignStatPlot R package offers an online implementation accessible at https://bioinformatics.um6p.ma/AlignStatPlot. This user-friendly platform employs interactive forms to facilitate the entire analysis pipeline. Users can input DNA sequences in Fasta format, along with an optional annotation file, and select their preferred sequence alignment tool (A). Moreover, for datasets consisting of fewer than 300 sequences (DNA or protein), users have the option to directly provide the sequence alignment, enabling the generation of circular format plots, which are particularly valuable (B). This feature enhances the visualization of sequence alignments, facilitating the exploration and analysis of the data.

**Fig 3:**
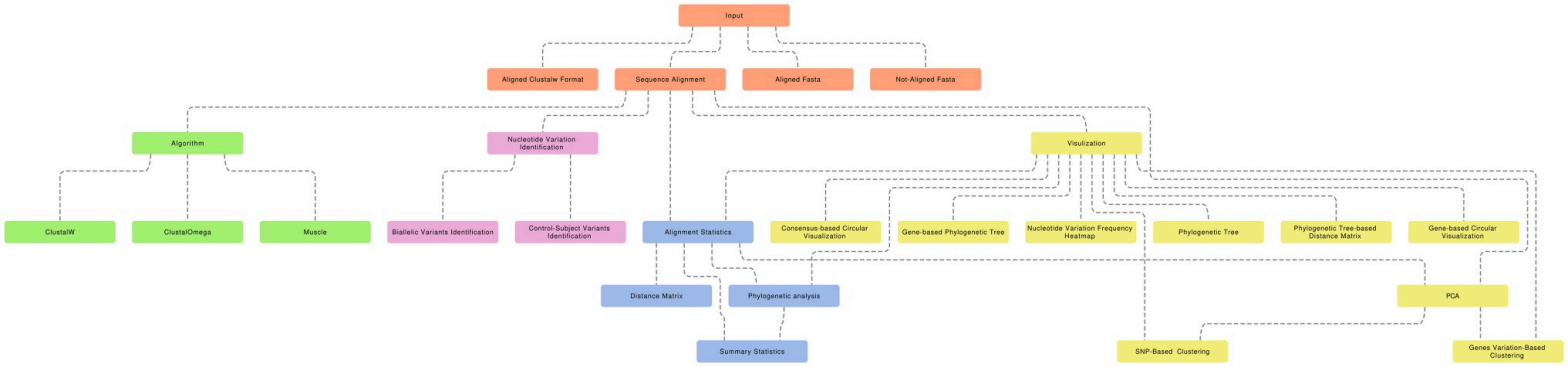
The AlignStatPlot flowchart illustrates the analysis workflow, showcasing the network of steps involved, as well as the possible input options and expected results and visualizations.

The proposed R package includes several comprehensive data analysis tools that allow users to perform sequence alignment, regular and innovative analyses, and data visualization (Fig 1). The tool was also made available to the public as an online tool to make it more accessible and user-friendly for researchers (Fig 2). AlignStatPlot was written primarily in the R programming language (98%), with 2% of the code written in C to increase the tool’s versatility with complex and large data sets. Using the online devtools R package, our R package can be easily installed from the github repository. Fig 3 depicts the total network of data analysis offered by the R package. AlignStatPlot performs sequence alignment analysis for DNA sequences in FASTA format. It can perform MSA analysis using Clustalw, ClustalOmega, [7] and Muscle [8]. The extracted sequence alignments are then formatted and used to provide a statistical overview of alignment performance and sequence similarity (Table 1). The R package circlize is used to provide an overview of sequence alignment [9] (Fig 1A and 1B). Classic visualizations, including phylogenetic and similarity matrices, are then automatically generated. When gene annotations are provided, combined plots are created to visualize possible shared aspects of sequence similarity, phylogenetic clusters, and gene structures (Fig 1C–1E). Matrices are generated to describe these variations, which are used for cluster analysis, including principal component analysis (PCA) across genes (Fig 1F). Nucleotide variations are then identified across sequences and large amounts of missing and non-biallelic nucleotides are removed to improve the next analysis procedures and focus on nucleotide variations that may contribute to gene evolution and diversity. SNP clustering is one of the new analyses introduced in the R package AlignStatPlot. The analysis uses filtered biallelic nucleotide variation and data clustering to detect possible nucleotide groups with correlated genetic variation across the sequences under consideration (Fig 1C–1E). This type of analysis may reveal a new way to study linkage disequilibrium phenomena at the gene level.

**Table 1:** Information about the sequences used to validate the AlignStatPlot package.

Data set	Genes	Study	Seq. count	Min. Len.	Max. Len.	Min. GC. Per.	Max. GC. Per.
Rice	COL4	[14]	130	993	999	73.67%	73.97%
	DPL1	[15]	117	853	854	38.57%	38.80%
	DTH7	[14]	125	2221	2268	47.29%	47.69%
	DTH8	[14]	136	875	897	70.06%	71.46%
	Ghd7	[14]	131	774	777	65.25%	66.02%
	Hd1	[14]	116	1181	1506	55.64%	59.11%
	Hd6	[14]	132	973	1002	42.42%	42.75%
	PhyB	[14]	125	3516	3516	52.19%	52.45%
	Se5	[14]	140	870	870	60.46%	60.57%
	LABA1	[16]	247	5029	5050	42.03%	42.13%
	TPP7	PopSet: 2106161045	475	2165	2320	58.06%	59.91%
	gammaTMT	PopSet: 2169220100	475	3300	3390	44.22%	44.75%
Maize	KRN2	PopSet: 2214448971	176	4709	5186	49.35%	50.60%
BRCA	BRCA2_1	[17]	28	5002	5002	34.23%	34.37%
	BRCA2_2	[18]	57	668	1184	23.96%	34.80%
	BRCA2_3	[19]	57	758	894	35.60%	39.51%
	BRCA2_4	[20]	72	696	864	31.72%	33.80%
	BRCA2_5	[21]	134	943	4359	31.47%	47.87%

For simplicity, users can do all the above-discussed steps with just one function called “AlignStatPlot”. In addition, users have the ability to specify their own analysis with a variety of built-in functions, all of which are well documented. Users can also visualize the sequence alignment by selecting the second option, which requires only the FASTA or clustalw sequence alignment format of the data. This step is accessible via the plotAlignCircle function or the online implementation (Fig 2). The online implementation of AlignStatPlot is hosted on a LAMP server running Linux 5.4.0–89-generic x86 64 (Ubuntu 20.04.3 LTS), Apache (version 2.4.41), and the R (v4.1.2) compiler. The LAMP server is powered by a machine with 32 GB of memory, 16-core CPUs and a 10 TB hard drive. HTCondor (v9.5.0) is used to manage and schedule tasks and processes. When the server’s job queue is full, jobs are routed to Africa’s fastest high-performance computer (TOUBKAL-POWEREDGE C6420, CRC-STACKHPC, XEON PLATNIUM 8276L 28C 2.2GHZ, MELLANOX INFINIBAND HDR100).

### Using PCA analysis for exploring the genetic data

Principal Component Analysis (PCA) is a widely used approach for investigating correlations among samples within a dataset. AlignStatPlot provides two distinct PCA plots to facilitate this analysis, utilizing the genetic variation data obtained from the PCA analysis. The first PCA plot delves into the relationship between samples, offering valuable insights into genetic diversity. By examining the interrelatedness of samples, we gain a deeper understanding of their genetic makeup and evolutionary history (Fig 1F). The second PCA plot specifically focuses on the detected Single Nucleotide Polymorphisms (SNPs) across genes. Within this plot, it is possible to observe the clustering of different SNPs into groups across the studied genes. These SNP clusters may exhibit similarities in terms of their location, variation, or inheritance patterns, shedding light on potential functional connections (Fig 1H). AlignStatPlot further augments the traditional PCA plots by providing an additional SNP clustering analysis plot. This plot not only pinpoints the location of shared SNPs but also reveals the specific groups to which they belong. By integrating this plot with the phylogenetic tree generated through MSA analysis, we gain deeper insights into the gene structure and annotation, enriching our understanding of the dataset (Fig 1I). This comprehensive approach enables us to capture both the genetic relationships among samples and the significance of SNP clusters within the gene context. It allows for meaningful comparisons with gene structure, annotation, and phylogenetic information, fostering a more comprehensive analysis of the dataset.

## Case study

We processed multiple gene sets to validate and demonstrate the potential use of AlignStatPlot in the fields of medicine, microbiology, and plant science (Table 1). We analyzed sequence data from several studies focused on the gene *BRCA1*, which regulates breast cancer progression. We included genes important to plant sciences, such as COL4 [10], DPL1 [11], and DTH7 [12] in rice and KRN2 [13] in maize. In microbiology, 16S and 18S rRNA sequences have been used to demonstrate the utility of AlignStatPlot in the study of prokaryotic diversity, especially when large numbers of genes are studied.

## Results

**Fig 4:**
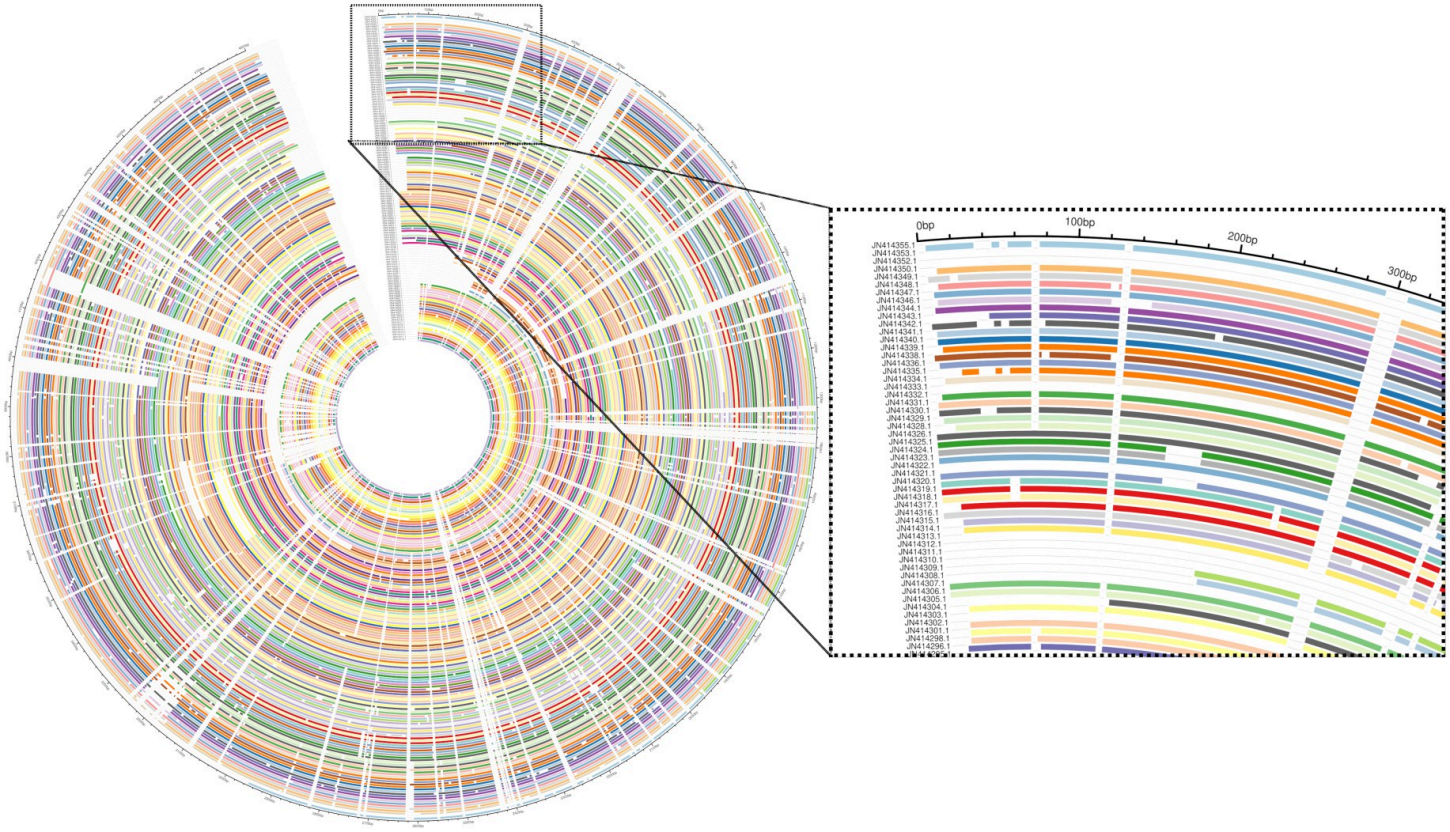
The sequence alignment visualization of large gene sets analyzed with the AlignStatPlot package.

Sequence alignment to study the genetic diversity of different genome samples is a common task for biology researchers. This task has become so overwhelming that tools and pipelines are required to obtain conclusive results and understandable, publication-ready figures. We introduce “AlignStatPlot,” a new R package and online tool that is well-documented and easy-to use for MSA and post-MSA analysis. This tool performs both traditional and cutting-edge analyses on sequencing data and generates new visualisation methods for MSA results. We tested our tool on a variety of gene sets (Table 1). More than 3273 sequences were analyzed using AlignStatPlot (Table 1, and S1 Table). The length of the gene sequences ranged from 696 to 5050 bp. Both the online and local versions were useful in analyzing these sequences and provided the expected results (Fig 1 and Table 1). For large gene sets, AlignStatPlot generated plots that indicated indels and shared sequences between genes (Fig 4). In the *BRCA* gene, numerous indels were found and were produced by some groups of sequences sharing a particular region. This type of pattern is uncommon in barcoding genes like 16S and 18S rRNA, which may be why it is useful in studies of microbial diversity, where mostly SNPs are the key factor for isolate identification [22]. Our tool provides two types of similarity matrices, one clustering genes based on their correlation of genetic variation and the other based on their order in the phylogenetic tree (S1 and S2 Figs). Both plots provide two distinct views. While clustering genes based on genetic variation provides a population structure-like view, phylogenetic ordering allows researchers to estimate the rate of similarity of clustered genes to other genes and determine how much similarity exists within and between genes (S3 and S4 Figs). Similar methods have been used to study the genetic diversity and evolution of viruses by comparing viral sequences of different historical strains using MSA and clustering analysis [23]. This method is widely used in gene-based sequencing to study gene diversity in plants [24].

**Fig 5:**
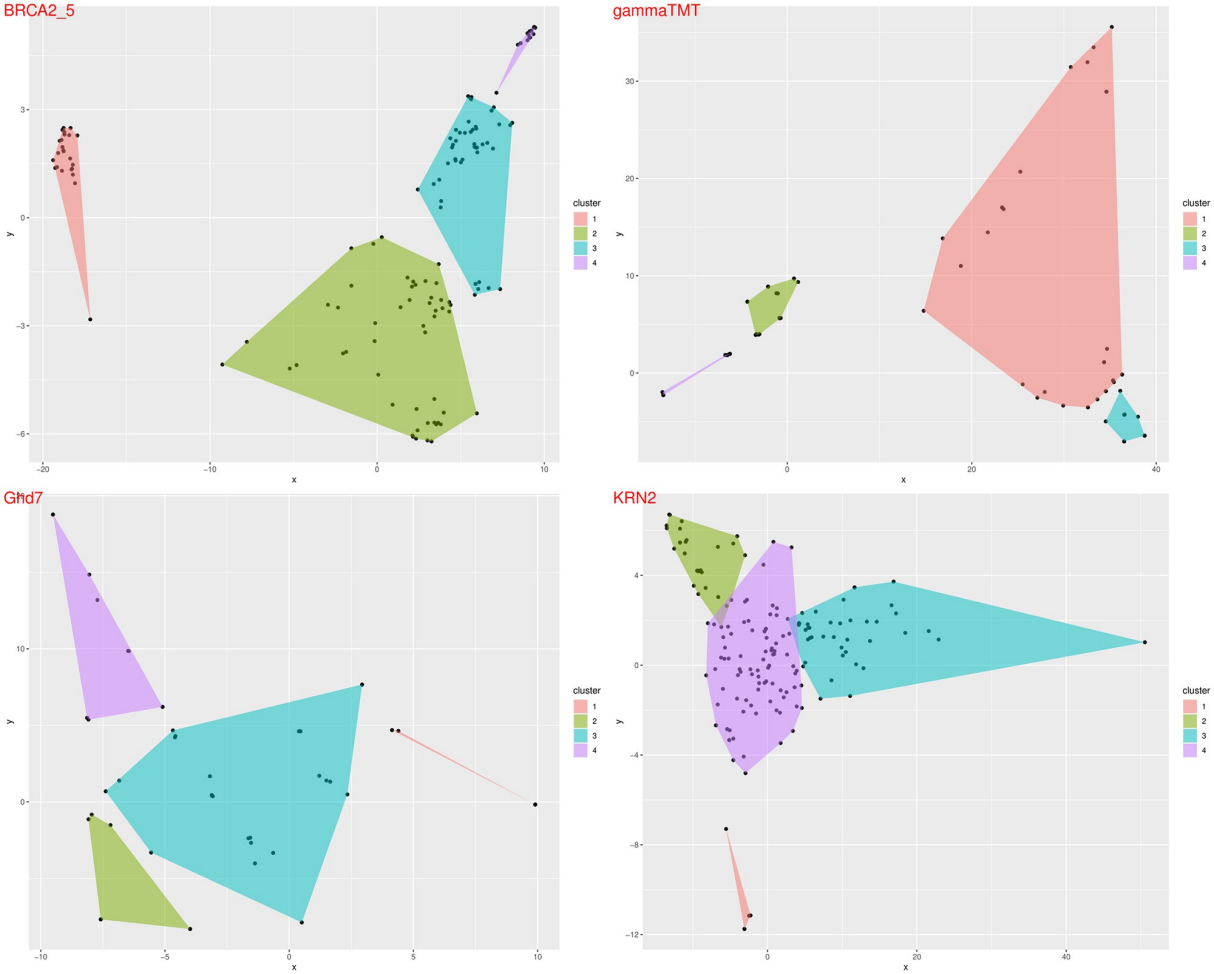
The PCA plot constructed for some genes with the AlignStatPlot package based on the findings of the MSA analysis of genetic variation.

PCA analysis is a common strategy for studying correlated genetic elements in samples. Gene clustering using PCA is another method to show correlated genes in the same data set. This method is very useful for studying functional genes as well as for bacterial gene diversity analysis using 16S rRNA [25]. Two different PCA methods are available in AlignStatPlot, one for studying clustering of genes based on SNP variation and the other for studying clustering of SNPs based on variation in genes (Fig 1). Gene sets such as BRAC2 and KRN2 showed distinct groups in PCA clustering of genes based on SNP variation (Fig 5). Clustering of SNPs based on their variation in different studies is a new analysis provided by AlignStatPlot. It shows that SNPs are clustered across genes regardless of their location. Such an analysis could provide a linkage disequilibrium-like view of nucleotide variation, but at the gene scale rather than the genome scale (Fig 1).

**Fig 6:**
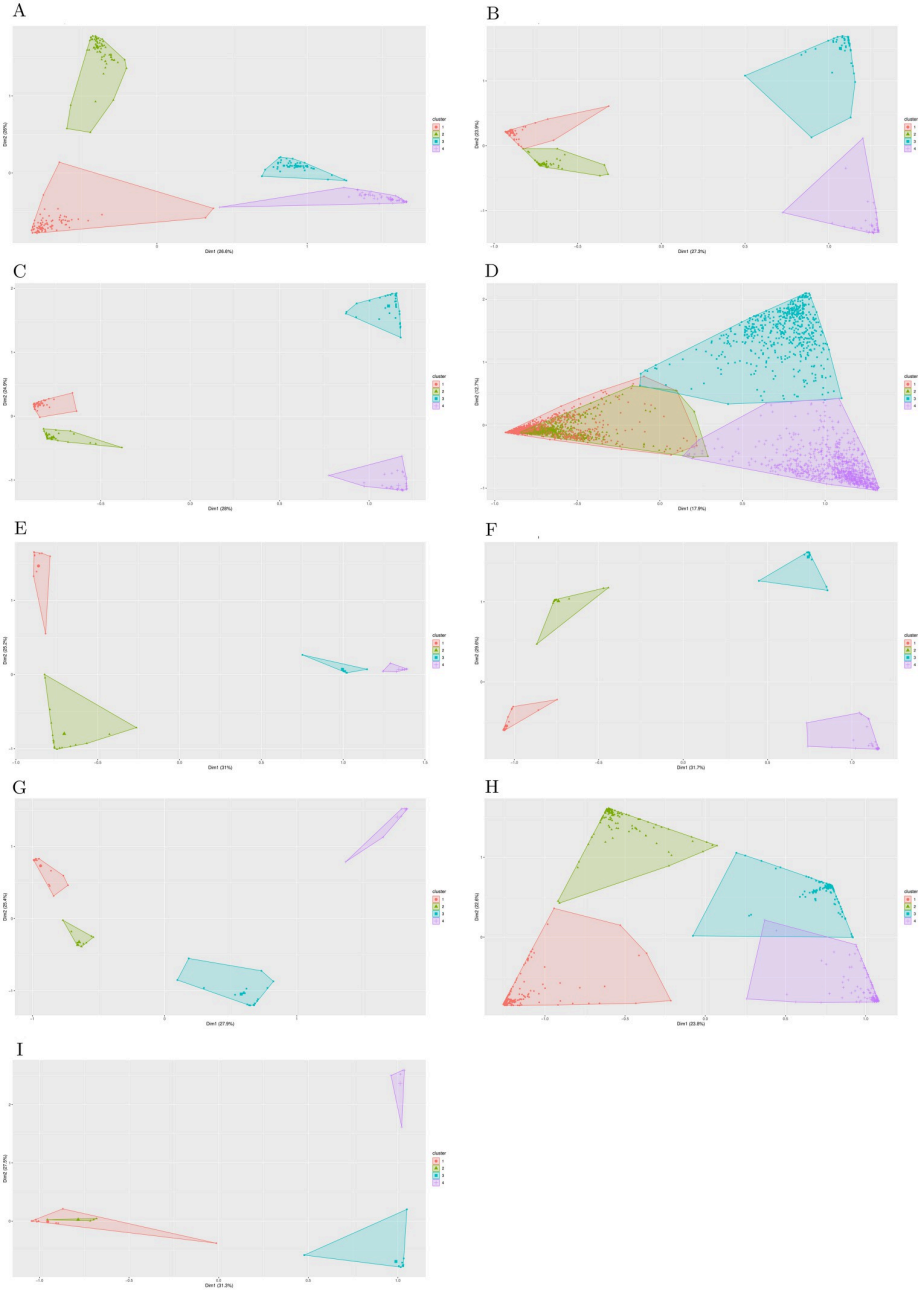
Based on the results of the MSA study, the PCA plot was created for certain SNPs using the AlignStatPlot tool.

Different SNP cluster groups were detected in the majority of the genes studied. These SNPs could be linked by their location, variation, or inheritance. Alignstaplot provides an additional plot for SNP clustering analysis that shows the location of these shared SNPs as well as the group to which they belong (Figs 1 and 6). This plot is combined with the phylogenetic tree generated by the MSA analysis to provide additional information about the analysis. SNP clustering analysis has been used previously to examine large groups and detect possible genome-level correlations [26, 27]. To our knowledge, such an analysis has never been used at the gene level, and it was also generated on the fly with minimal effort. AlignStatPlot will produce both a phylogenetic tree plot and a statistical summary file. If the annotation for the genes is provided, a phylogenetic tree with exons, introns, and other gene components will be generated (Fig 4). Which in useful in several studies such as gene family analyses [28]. The similar gene structure was reflected by the phylogenetic clustering in some of the investigated genes (Fig 4). The supplementary data contains a detailed discussion of the validation data.

## Conclusion

AlignStatPlot has the potential to be successfully integrated in a variety of genomics fields, including medical, crop, and microbial genetics. The tool generated MSA analysis methods that were both traditional and advanced. It includes several analysis procedures that make use of the MSA analysis output in an easy-to-use manner. The tool can be easily combined with several population genetics tools that process bi-allelic data. The online tool will make it easier for researchers who do not have a programming background to produce publishable results.

## Supporting information

S1 Table An overview of the data analysis. An overview of the data analysis findings that were used to verify the alignstatplot tool.
(DOCX)

S1 Fig Sequence similarity matrix of the studied case study data. Correlation of sequence similarity generated using MSA analysis.
(JPG)

S2 Fig Sequence similarity matrix of the studied case study data with tree of group A. Correlation of sequence similarity generated using MSA analysis combined with phylogenetic tree group A.
(JPG)

S3 Fig Sequence similarity matrix of the studied case study data with tree of group B. Correlation of sequence similarity generated using MSA analysis combined with phylogenetic tree group B.
(JPG)

S4 Fig Phylogenetic tree combined with with gene structure. Phylogenetic tree combined with with the gene structure of some of the case studied data.
(JPG)

S5 Fig (JPG)
