# Supplementary material for: AlignStatPlot: An R package and online tool for robust sequence alignment statistics and innovative visualization of big data
Source: PLoS One. 2023 Sep 20;18(9):e0291204. doi: 10.1371/journal.pone.0291204 (PMC10511070; doi:10.1371/journal.pone.0291204)
Supplement: S1 Table — An overview of the data analysis findings that were used to verify the alignstatplot tool. (DOCX) [file pone.0291204.s001.docx]

Table 1: An overview of the data analysis findings that were used to verify the alignstatplot tool.

Phylogenetic analysis

| Data set | Genes | Alignment | |  | | | | | | | | |
| --- | --- | --- | --- | --- | --- | --- | --- | --- | --- | --- | --- | --- |
|  |  |  |  | Branch lengths | | | | Distribution summary | | | | |
|  |  | Min. Gap Per. | Max. Gap Per. | Tips | nodes | mean | variance | Min. | 1stQu. | Median | 3rdQu. | Max. |
| Rice | COL4 | 0.00% | 0.60% | 130 | 128 | 0.00174 | 2.70E-05 | -5.91E-05 | 0.00E+00 | 0.00E+00 | 4.40E-18 | 3.95E-02 |
|  | DPL1 | 0.00% | 0.12% | 117 | 115 | 0.00041 | 1.08E-05 | -6.65E-07 | -7.03E-24 | 0.00E+00 | 1.05E-22 | 3.67E-02 |
|  | DTH7 | 0.00% | 2.07% | 125 | 123 | 0.00273 | 3.01E-05 | -4.26E-05 | 0.00E+00 | 4.00E-18 | 1.55E-03 | 2.90E-02 |
|  | DTH8 | 0.66% | 3.10% | 136 | 134 | 0.00153 | 4.27E-05 | -2.05E-03 | -4.59E-14 | 0.00E+00 | 1.00E-09 | 6.03E-02 |
|  | Ghd7 | 0.38% | 0.77% | 131 | 129 | 0.00429 | 7.95E-05 | -3.52E-17 | 0.00E+00 | 0.00E+00 | 1.85E-03 | 4.41E-02 |
|  | Hd1 | 0.20% | 21.74% | 116 | 114 | 0.00398 | 8.71E-05 | -8.27E-03 | -1.48E-11 | 7.39E-11 | 1.50E-03 | 5.74E-02 |
|  | Hd6 | 0.00% | 2.89% | 132 | 130 | 0.00098 | 1.77E-05 | -2.19E-06 | -2.21E-24 | 2.89E-36 | 1.61E-18 | 2.23E-02 |
|  | PhyB | 0.00% | 0.00% | 125 | 123 | 0.00166 | 2.02E-05 | -3.48E-17 | 0.00E+00 | 0.00E+00 | 4.09E-18 | 3.77E-02 |
|  | Se5 | 0.00% | 0.00% | 140 | 138 | 0.00021 | 4.48E-06 | -5.20E-18 | 0.00E+00 | 0.00E+00 | 0.00E+00 | 2.40E-02 |
|  | LABA1 | 0.59% | 1.00% | 247 | 245 | 0.00063 | 8.12E-06 | -6.60E-03 | -1.44E-21 | 1.31E-41 | 1.44E-11 | 2.93E-02 |
|  | TPP7 | 10.13% | 9.60% | 475 | 473 | 0.00089 | 1.97E-05 | -8.18E-05 | -4.57E-60 | 1.19E-109 | 3.91E-39 | 7.59E-02 |
|  | gammaTMT | 0.91% | 3.54% | 475 | 473 | 0.00114 | 2.07E-05 | -5.76E-06 | -2.42E-35 | 1.30E-74 | 2.03E-16 | 5.39E-02 |
| Maize | KRN2 | 17.58% | 25.16% | 176 | 174 | 0.02212 | 0.00060 | -0.00029 | 0.00195 | 0.00697 | 0.04513 | 0.13852 |
| BRCA | BRCA2_1 | 0.00% | 0.00% | 28 | 26 | 0.00572 | 2.56E-05 | -4.27E-18 | 1.86E-03 | 4.96E-03 | 8.03E-03 | 2.43E-02 |
|  | BRCA2_2 | 0.00% | 9.04% | 57 | 55 | 0.02874 | 0.00222 | -0.00592 | 0.00320 | 0.01592 | 0.03965 | 0.42409 |
|  | BRCA2_3 | 0.33% | 7.80% | 57 | 55 | 0.02675 | 0.00078 | -0.00046 | 0.00821 | 0.02385 | 0.03497 | 0.16031 |
|  | BRCA2_4 | 13.26% | 4.53% | 72 | 70 | 0.01753 | 0.00058 | -0.00122 | 0.00068 | 0.01432 | 0.02556 | 0.21071 |
|  | BRCA2_5 | 10.40% | 80.62% | 134 | 132 | 0.08361 | 0.00562 | 0.00046 | 0.01140 | 0.06963 | 0.13766 | 0.32638 |

1
