# Supplementary figures and images for: AlignStatPlot: An R package and online tool for robust sequence alignment statistics and innovative visualization of big data
Source: PLoS One. 2023 Sep 20;18(9):e0291204. doi: 10.1371/journal.pone.0291204 (PMC10511070; doi:10.1371/journal.pone.0291204)

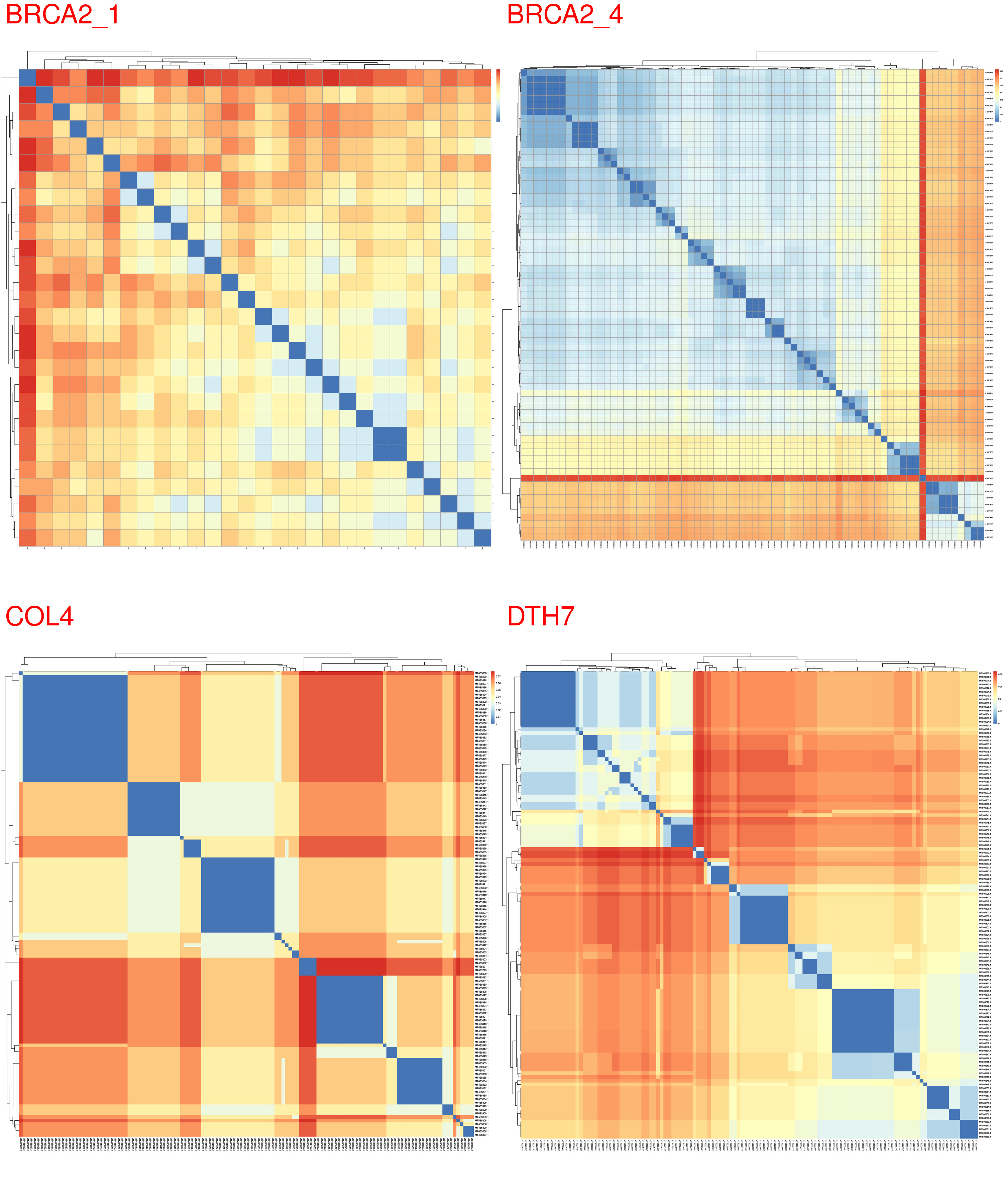

Supplement: S1 Fig — Correlation of sequence similarity generated using MSA analysis. (JPG) [file pone.0291204.s002.jpg]

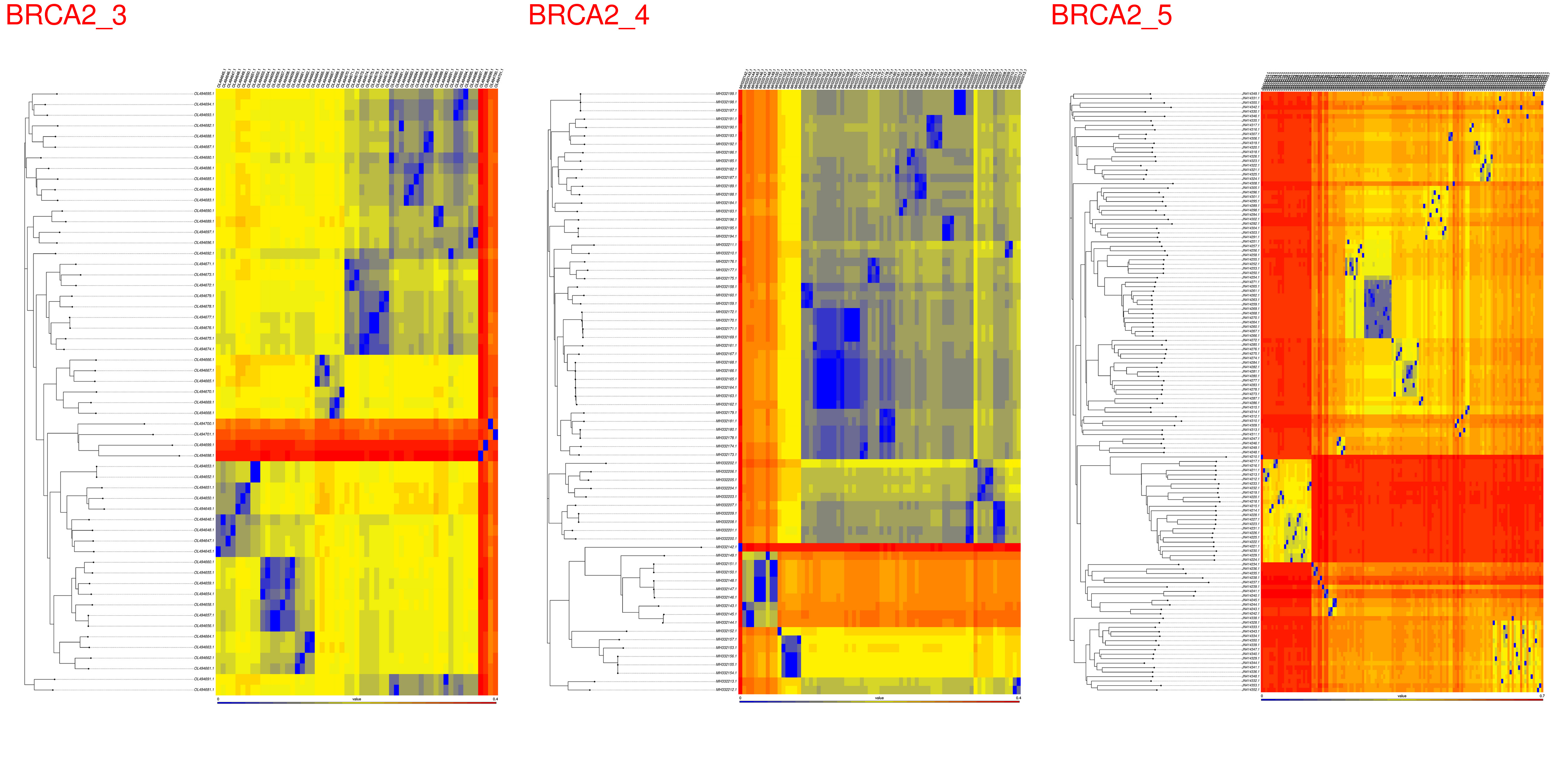

Supplement: S2 Fig — Correlation of sequence similarity generated using MSA analysis combined with phylogenetic tree group A. (JPG) [file pone.0291204.s003.jpg]

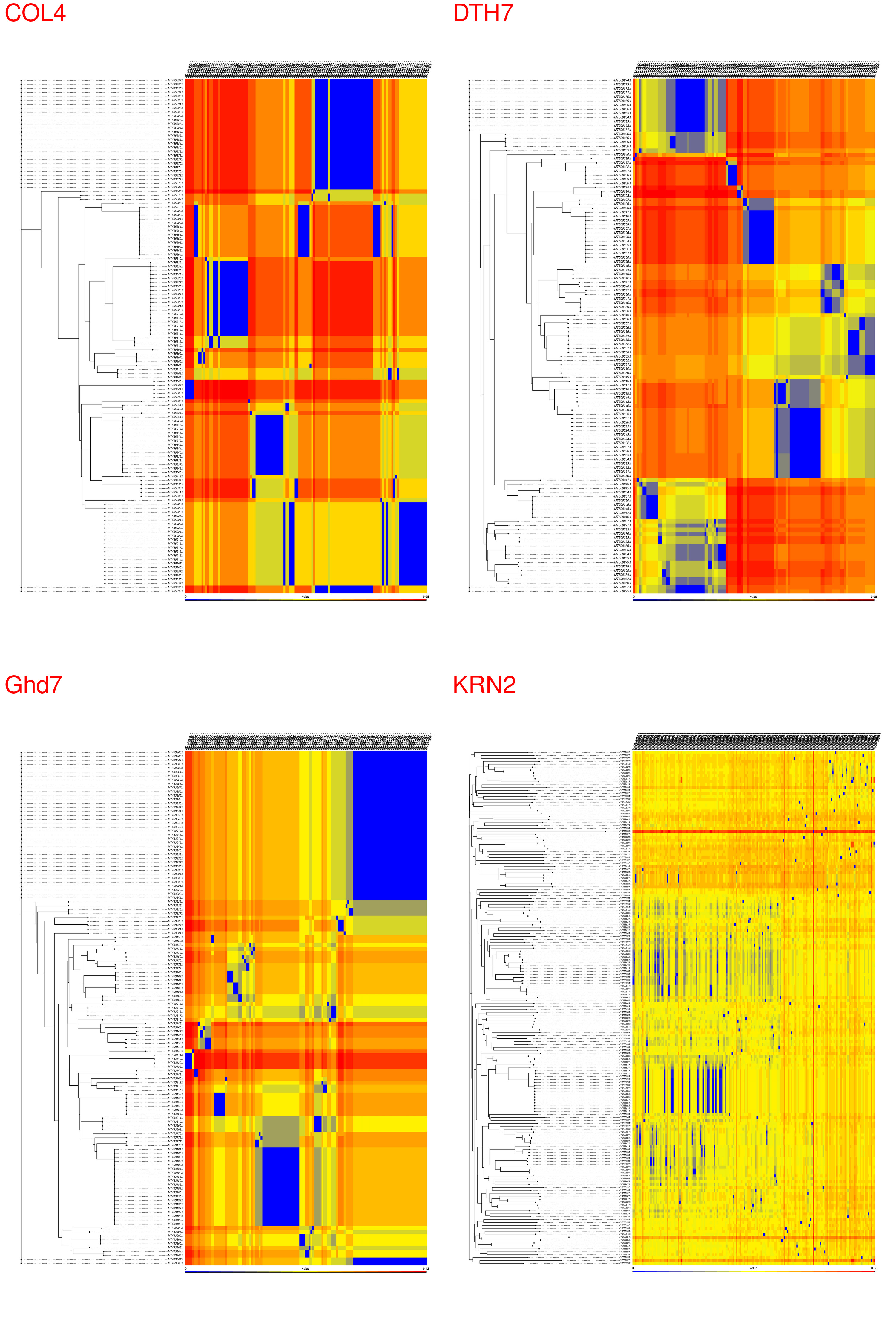

Supplement: S3 Fig — Correlation of sequence similarity generated using MSA analysis combined with phylogenetic tree group B. (JPG) [file pone.0291204.s004.jpg]

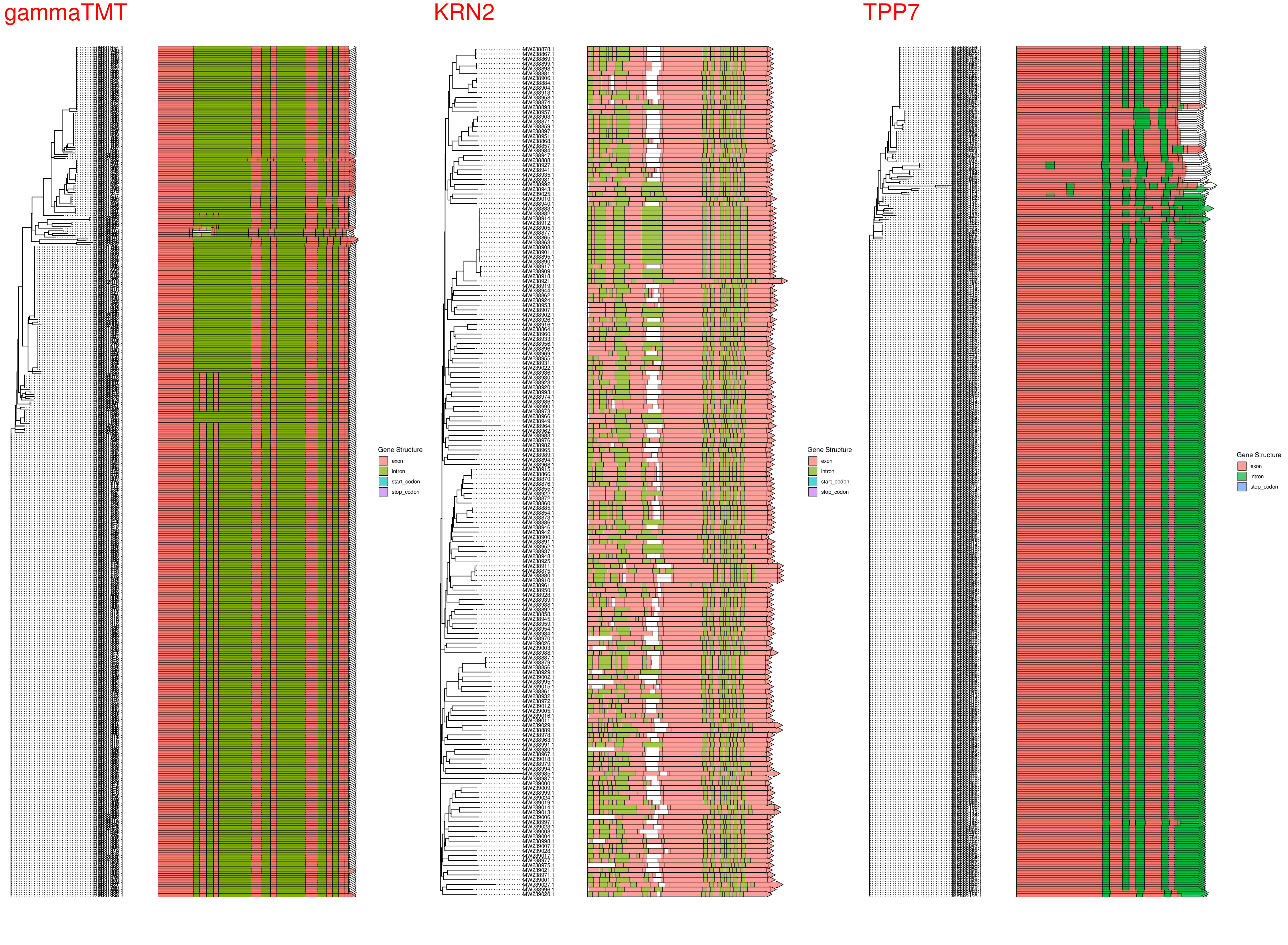

Supplement: S4 Fig — Phylogenetic tree combined with with the gene structure of some of the case studied data. (JPG) [file pone.0291204.s005.jpg]

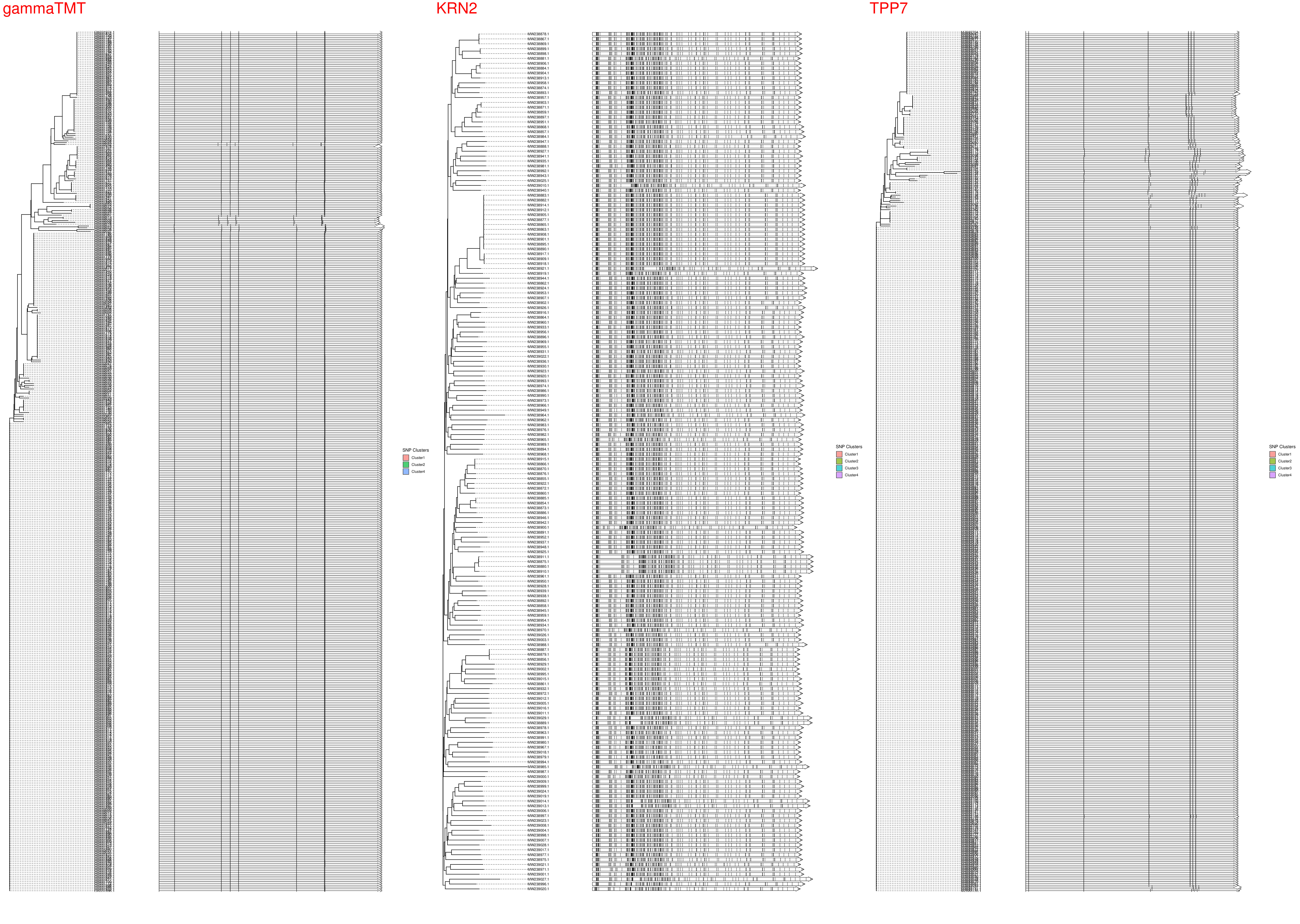

Supplement: S5 Fig — (JPG) [file pone.0291204.s006.jpg]
